# Modulation of c-Fos and BDNF Protein Expression in Pentylenetetrazole-Kindled Mice following the Treatment with Novel Antiepileptic Compound HHL-6

**DOI:** 10.1155/2014/876712

**Published:** 2014-01-29

**Authors:** Saima Mahmood Malhi, Huma Jawed, Farina Hanif, Nadeem Ashraf, Farhat Zubair, Bina S. Siddiqui, Sabira Begum, Nurul Kabir, Shabana Usman Simjee

**Affiliations:** ^1^H.E.J. Research Institute of Chemistry, International Center for Chemical and Biological Sciences, University of Karachi, Karachi 75270, Pakistan; ^2^Dr. Panjwani Center for Molecular Medicine and Drug Research, International Center for Chemical and Biological Sciences, University of Karachi, Karachi 75270, Pakistan

## Abstract

Brain-derived neurotrophic factor (BDNF) and c-Fos are shown to promote epileptogenesis and are taken as a marker of neuronal activity. The present study investigated the expression of BDNF and c-Fos in mice brain with pentylenetetrazol- (PTZ-) induced generalized seizure and evaluated the effect of novel tryptamine derivative HHL-6 on the expression of these two markers. The subconvulsive dose of PTZ (50 mg/kg) was administered on alternate days in the experimental groups until the seizure scores 4-5 developed in the PTZ-control group. At the end of each experiment, animals were sacrificed, brain samples were collected and cryosectioned, and immunohistochemical analysis of BDNF and c-Fos protein was performed. Data obtained from two sections per mouse (*n* = 12 animals/group) is presented as means ± S.E.M. The test compound HHL-6 demonstrated a potent anticonvulsant activity in the PTZ-induced seizure in mice. Significant reduction in the BDNF (*P* < 0.003) and c-Fos (*P* < 0.01) protein expression was observed in the HHL-6 treated group. Based on these results we suggest that one of the possible mechanisms of HHL-6 to inhibit epileptogenesis might be due to its controlling effect on the cellular and molecular expression of the factors that contribute to the development of epileptogenic plasticity in the CNS.

## 1. Introduction

Epilepsy is a neurological disorder manifested by rapid and recurrent seizures, resulting from synchronized discharges of neurons in brain, affecting at least 1% of the world population [1, 2]. To explain the mechanism how seizures are generated in specific brain areas or different brain structures, various studies have been performed worldwide involving behavioral, electrophysiological, and molecular analysis [3–6]. So far, the immense majority of epilepsy research has been executed in rodents [7–10] and stimulation of neurons by electrical or chemical induction resulting in seizure discharges is believed to be the most common way to contribute to the process of epileptogenesis referred to as “kindling” [11, 12].

In kindling, an initially subconvulsive chemical or electrical convulsant stimulus of the brain evokes seizure discharges. The molecular mechanisms underlying the development of the abnormal excitability in kindling or epilepsy are poorly understood. However, it is believed that structural rearrangements responsible for maintenance of epileptic syndrome might be triggered by trophic factors such as BDNF (brain derived neurotrophic factor) that is involved in various signaling mechanisms that may be influenced by seizure activity [13]. Among various trophic factors, much interest has been focused on the BDNF, NGF, and NT-3 and their high-affinity receptors TrkA, TrkB, and TrkC. A variety of epilepsy models have shown that seizure activity induces transient changes of neurotrophin gene expression in neurons [14–17]. In addition to these trophic factors, the proto-oncogene c-Fos has also been shown to be induced transiently in response to various stimuli such as seizures induction [18]. The c-Fos gene encodes a DNA-binding protein, c-Fos, which forms a heterodimeric transcriptional factor, activator protein-1 (AP-1), by direct participation or with c-jun [19, 20]. The activation of c-Fos expression in neurons by seizures led to its proposed use as a marker of neuronal activity [21–23].

In the present study, we have used pentylenetetrazole (PTZ) to induce epileptogenesis in mice. The reason for using this chemoconvulsant was based on the studies reporting that the PTZ dependent induction of c-Fos expression might play an important role in the development of seizures and excitatory amino-acid induced toxicity. Thus, during the development of kindling, each stimulus-evoked seizure leads to increased expression of BDNF and c-Fos resulting in seizure development [24, 25]. Due to the fact that BDNF and c-Fos expression in neurons is transiently increased during seizures, we have used them as molecular indicators of neuronal activity in various brain regions to monitor the effect of our test compound, HHL-6.

## 2. Methodology

### 2.1. Animals

Albino male NMRI mice weighing 20–25 grams were used. The animals were housed under standard laboratory conditions, that is, 12 : 12 hr light : dark cycle, temperature (21 ± 2°C), and air humidity controlled environment. Food and water were provided *ad libitum*. All experiments were carried out in accordance with the International Guidelines for the Use and Care of Laboratory Animals and approved by Scientific Advisory Committee on Animal Care, Use, and Standards of our institute.

### 2.2. Drugs or Test Compound

**Figure 1:**
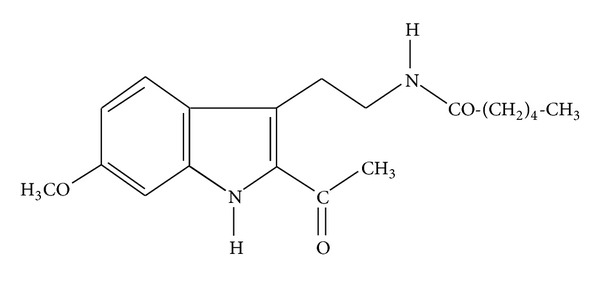
Structure of tryptamine derivative HHL-6 (2-acetyl-3-(2-hexanoylamidoethyl)-7-methoxyindole).

Pentylenetetrazole (PTZ) was used as a chemical convulsant whereas diazepam was used as a reference drug. The test compound HHL-6 (Figure 1) was synthesized by our chemist collaborator at H.E.J. Research Institute of Chemistry, International Center for Chemical and Biological Sciences. Since the chemical convulsant PTZ is photosensitive, therefore, care was taken to prepare fresh PTZ solution on the day of administration without unnecessary exposure to the light. Primary antibodies, that is, purified c-Fos (4): sc-52 and BDNF (N-20): sc-546, were purchased from Santa Cruz Biotechnology (USA) and secondary antibody Alexa flour 488 goat antimouse IgG(H + L)^∗2^ was purchased from Invitrogen (USA).

### 2.3. *In Vivo* Assessment of Anticonvulsant and Antiepileptic Activities of HHL-6

Tables 1 and 2 outline the treatment regime followed for both the anticonvulsant and antiepileptic activity testing of HHL-6.

#### 2.3.1. Subcutaneous-PTZ-Induced Seizure Test

Anticonvulsant effect of HHL-6 using scPTZ test has been evaluated by administering 20 mg/kg dose to groups of 6 mice at least 30 min before subcutaneous administration of convulsive dose of PTZ (90 mg/kg). After PTZ administration, animals were isolated and observed for an hour for the presence or absence of different types of seizure patterns. Latency to PTZ-induced threshold seizures was also calculated. The latency to threshold seizure is defined as the interval between the time of the PTZ injection and the occurrence of the first episode of threshold seizure. Protection of testing material against PTZ-induced mortality within 24 hours was also evaluated. In all experiments, diazepam (7.5 mg/kg i.p) was used as positive control.

#### 2.3.2. scPTZ-Induced Kindling (Antiepileptogenic Activity)

Subconvulsive dose (50 mg/kg) of PTZ was given subcutaneously to all groups (*n* = 12/group) except for normal control after every 48 hours. In normal control group, instead of PTZ, saline was used. The drug control groups received daily administration of diazepam (7.5 mg/kg). The test group was treated with the HHL-6 (20 mg/kg) once daily. Racine's scale was used to score the seizure activity such as stage 0: no response; stage 1: ear and facial twitching; stage 2: convulsive wave through the body; stage 3: myoclonic jerks, stage 4: clonic-tonic convulsions, turnover into side position; stage 5: generalized clonic-tonic seizures, loss of postural control. Animals showing score 4 or 5 were considered to be fully kindled.

#### 2.3.3. Behavioral Analysis

Animals were transferred into individual cages the day before the experiments to allow them to acclimatize to the new environment. Animals were observed in these cages for 1-2 hr after drug treatment. Behavior (locomotion, head weaving, biting, licking or grooming, hyper-excitability, ataxia and sedation, writhing, jumping, etc.) of the animals was observed for 1-2 hr after they were injected with vehicle, standard drug, and test sample. For behavioral studies, blind testing was employed; that is, the experimenter conducting this study was blinded to the given treatment.

#### 2.3.4. Muscle Relaxant Activity

This was examined by traction test. The forepaws of a mouse were placed on a small twisted wire rigidly supported above a bench top. Normal mice grasped the wire with forepaws and, when allowed to hang free, placed at least one hind foot on the wire within 5 seconds. Inability to put up at least one hind foot constituted failure to the traction. The test was conducted at 30 min and 1 h after the injection of saline, diazepam, or HHL-6.

### 2.4. Sample Collection and Tissue Preparation

Animals were sacrificed 24 hrs after establishment of complete kindling. Cardiac perfusion with chilled PBS buffer (NaCl—8 g, KCl—0.2 g, Na_2_HPO_4_ × 12 H_2_O—2.85 g, and KH_2_PO_4_—0.2 g; pH 7.4) containing heparin was performed. Whole brains were removed, postfixed in ice-cold 2% PFA for 24 hrs at +4°C, and then placed in 30% sucrose until brain samples sank to the bottom of the container. The samples were stored at −80°C until further processing.

### 2.5. Immunohistochemistry for c-Fos and BDNF Protein Expression

Frozen brain samples were cut into 30 *μ*M thick sections and directly taken on poly L-lysine coated slides and kept at −20°C overnight. The cryosections were incubated overnight at 4°C with primary antibody, that is, purified c-Fos (4): sc-52 for c-Fos detection and BDNF (N-20): sc-546 for BDNF detection independently. This was followed by reincubation with secondary antibody, that is, biotinylated goat anti-mouse IgG (Alexa Flour) for 1 hour at room temperature in dark. The stained sections were observed under fluorescent microscope (Nikon ECLIPSE TE2000-S, Japan). Since the specificity of the primary antibody is very critical therefore, in order to determine specificity, negative controls were also processed by eliminating the step, where sections were incubated with their respective primary antibody.

Expression of c-Fos and BDNF protein observed under the fluorescent microscope was analyzed by using software *ImageJ* (NIH, USA). The image processing program *ImageJ* helps in multiple imaging system data comparisons taking density (densitometry) into consideration to analyze the images. For each image, background density was determined and subtracted; the remaining particles were considered to represent c-Fos or BDNF expression. Data were obtained from two sections per mouse (*n* = 12 animals per group) and presented as means ± S.E.M. cFos and BDNF immunoreactivity in the amygdala, cortex, dorsal hippocampus, and thalamus were centered approximately around 3.6 mm posterior to Bregma. Within the hippocampus region, measurements were performed over the layer extending from subregions CA1–CA3.

### 2.6. Statistical Analysis

Data is presented as mean ± standard error of the mean (S.E.M.). The seizure activity was analyzed by nonparametric Mann-Whitney *U* tests. The immunostaining data was analyzed by one-way ANOVA with post hoc Dunnett's multiple comparison tests. Sequential differences among means were calculated at the level of *P* < 0.05 using the SPSS version 10.

## 3. Results

### 3.1. scPTZ-Induced Seizure Test and PTZ-Induced Mortality

**Figure 2:**
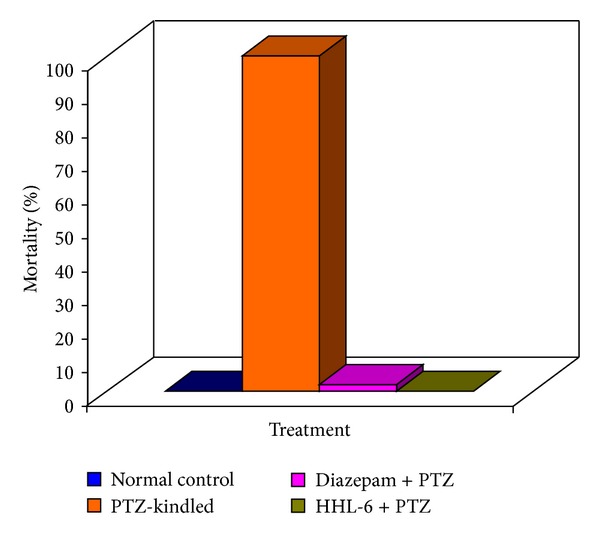
Percent mortality in different treated groups. Animals in the diazepam and HHL-6 treatment groups demonstrated 100% protection against scPTZ-induced mortality.

The HHL-6 was unable to provide protection from jerks; however, the seizure behavior did not progress further. Moreover, the latency to the onset of jerks was also delayed compared to PTZ-control group. No rearing or falling was observed in the HHL-6 treated group compared to PTZ-control group (Table 3) and 100% protection from tonic fore limb and hind limb seizures was observed. In contrast, in PTZ-control group, once seizures were started these were more frequent and severe. In scPTZ test, 100% mortality was observed in PTZ-control animals only. Mortality was not demonstrated by groups receiving 20 mg/kg of HHL-6 or 7.5 mg/kg of diazepam (Figure 2).

### 3.2. Subcutaneous-Pentylenetetrazole- (scPTZ-) Induced Kindling in Mice

**Figure 3:**
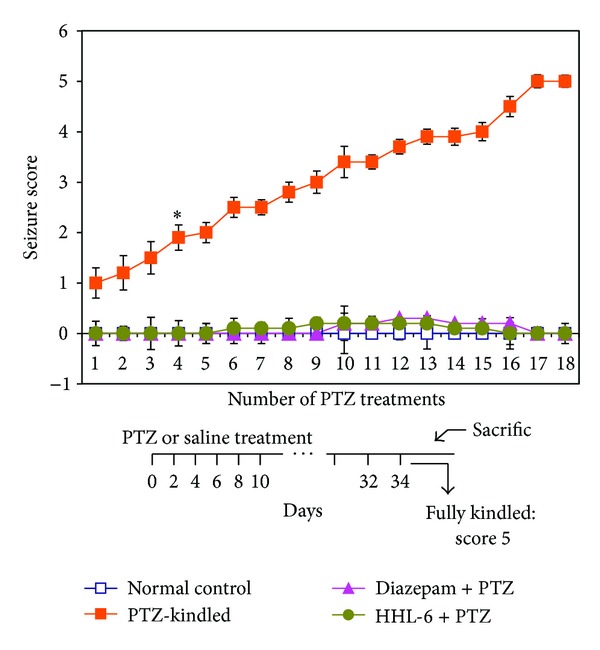
Effect of HHL-6 on scPTZ-induced kindling. The kindling scores are expressed as the arithmetic mean ± SEM (*n* = 12/group). The treatment of 20 mg/kg of HHL-6 showed a slight increase in their seizure score from treatment day 9 to 13 and thereafter it returned to the 0. The bar under the figure represents the timeline of the experiments. The mice were treated with PTZ or saline on alternate days. A total of 18 doses were given during the 36-day period when animals in control group were fully kindled. The average scores of PTZ-induced seizures were significantly higher in the kindled control mice (receiving no other treatment except for PTZ). *P < 0.05: significantly higher than normal control revealed by nonparametric Mann-Whitney *U* tests. The animals were sacrificed for immunohistochemistry and RT-PCR analysis after 36 days.

The animals in scPTZ-kindled control group displayed a gradual increase in the seizure score reaching score 5 after 18 treatments with an average seizure score of 4.9. Compared to PTZ-kindled control group, the diazepam treated group did not exhibit any seizure pattern till the end of the kindling protocol; however, a slight nonsignificant score of 0.3 was observed in case of HHL-6 group at the tested dose during treatment day 9 till 13; thereafter, it completely retarded the development of kindling induced by scPTZ administration (Figure 3). Within the treatment groups, no difference was found in the diazepam and HHL-6 treated animals.

### 3.3. Behavioral Analysis and Muscle Relaxant Activity

Animals treated with HHL-6 did not show any alteration in their normal behavior pattern. Hyperexcitability or sedation was also not observed in the HHL-6 treated group.

The muscle relaxant activity was tested by traction test. We did not observe muscle relaxant activity of HHL-6 (20 mg/kg) in the treated animals. Since we have used 20 mg/kg of HHL-6 in our kindling study therefore, in muscle relaxant activity analysis we did not increase the dose further.

### 3.4. c-Fos and BDNF Immunohistochemistry

**Figure 4:**
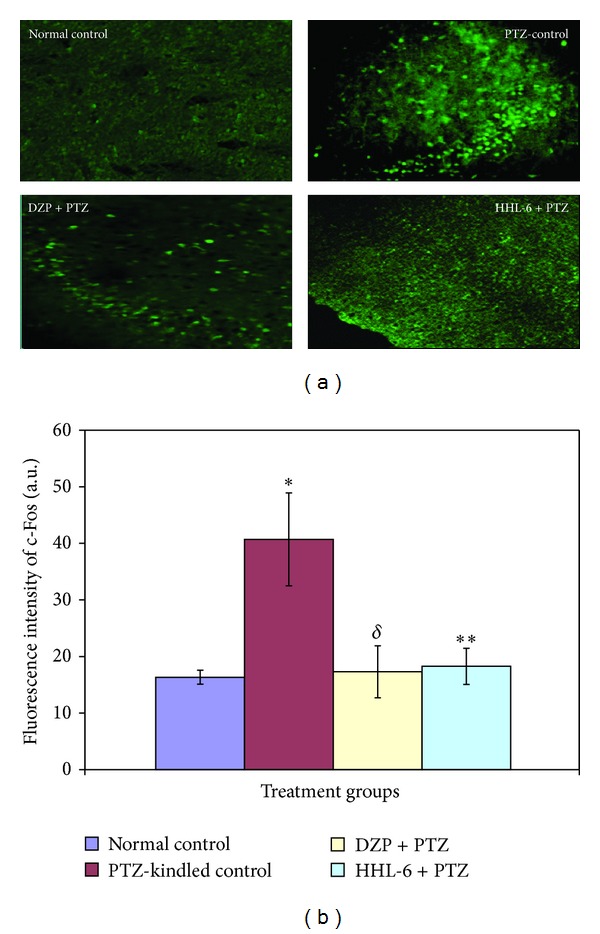
(a) Photomicrograph of c-Fos immunoreactivity in cortical region of normal and kindled mice brains with stage 5 seizure activity. Note the increased and scattered immunofluorescence in the PTZ-kindled control brain section. In contrast, the immunoreactivity of c-Fos in the brain of the kindled animals treated with HHL-6 and diazepam was markedly reduced. (b) Graphical representation of the level of expression of immunohistochemically detected c-Fos in kindled and unkindled brain sections analyzed by ImageJ software. In comparison to the normal control group (16.31 ± 1.24), the PTZ-control animals showed a significant increase in the c-Fos staining intensity (40.69 ± 8.2, *P < 0.001). In contrast, c-Fos expression in the kindled animals treated with HHL-6 (18.24 ± 3.2, **P < 0.01) and diazepam (17.28 ± 4.6, ^*δ*^ *P* < 0.05) declines significantly.

In the normal control group, there was little to no immunoreactivity of c-Fos and BDNF. Immunohistochemical analysis revealed a significant increase in the expression of c-Fos protein in the brain sections of control PTZ-kindled mice (*P* < 0.001, Figure 4) which was centered especially in the CA1-CA2 hippocampal region (3.6 mm posterior to the Bregma) and thalamus, when compared with the normal brain (Table 4). Within the cortex a relatively uniform distribution of cFos across all of the cortical layers was found. In contrast, the animals treated with diazepam (7.5 mg/kg, *P* < 0.05) showed a significant reduction in the expression of c-Fos protein which was almost comparable to the normal control group. Within the treatment groups, the brain sections from HLL-6 treated animals showed slightly more expression of c-Fos as compared to the diazepam treated kindled group; however, ANOVA with Dunnett's multiple comparison test analysis demonstrated that this difference was nonsignificant (*P* < 0.623).

**Figure 5:**
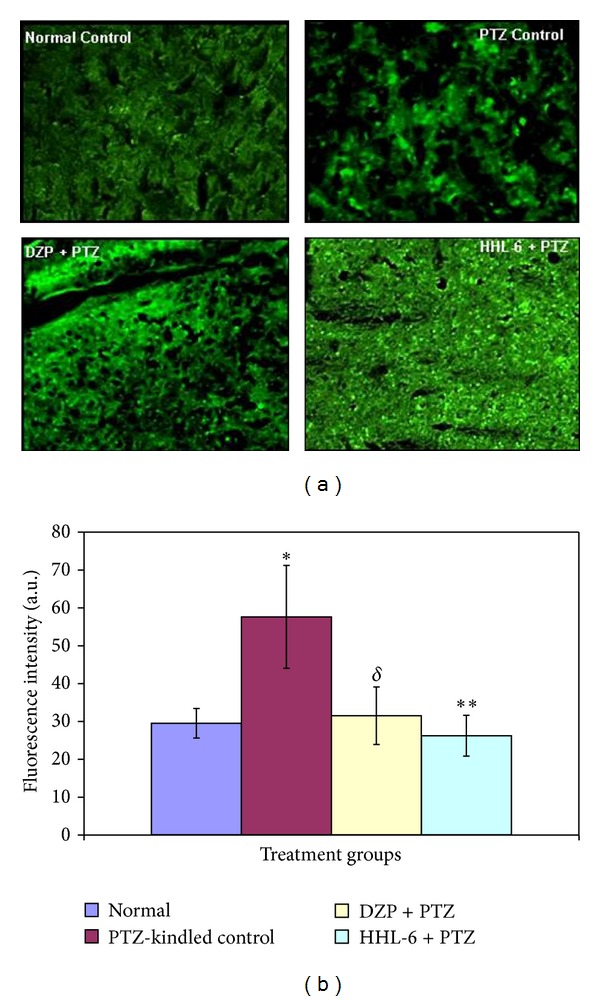
(a) Images of the BDNF immunoreactivity in cortical region of normal and kindled mice brains. A marked increase in the BDNF immunofluorescence was observed in the PTZ-kindled control brain section. In comparison to the PTZ-control group, the immunoreactivity of BDNF in the brain sections of the kindled animals treated with HHL-6 and diazepam was markedly reduced. (b) Graphical representation of the level of expression of immunohistochemically detected BDNF. The captured immunoimages were analyzed by ImageJ software. In comparison to the normal control group (29.53 ± 3.9), the PTZ-control animals showed a significant increase in the BDNF staining intensity (57.6 ± 13.6, *P < 0.001). In contrast, BDNF expression in the kindled animals treated with HHL-6 (26.2 ± 5.4, **P < 0.003) and diazepam (31.5 ± 7.6, ^*δ*^ *P* < 0.02) declines significantly.

The BDNF immunohistochemical analysis of the brain sections from normal control animals reveals low levels of BDNF protein (Figures 5(a) and 5(b)). This distribution was indicative of BDNF protein-like immunoreactivity found in the mouse brain. In comparison to the normal control group, the PTZ-kindled control group has shown a marked increase in the expression of BDNF protein (*P* < 0.001). Conversely, intriguing results were seen by our test compound HLL-6 (a tryptamine derivative) on the expression of BDNF protein as compared to standard anti-epileptic drug such as diazepam in PTZ-kindled mice. The ANOVA analysis demonstrated that the reduction in the BDNF in HHL-6 treated group was significantly different from PTZ-kindled control group (*P* < 0.00).

## 4. Discussion

Well-defined seizure behaviors in kindling were initially reported and clearly illustrated by Racine and his team [26, 27]. The systemic administration of PTZ results in well-established generalized tonic-clonic seizures providing a good evidence to study epileptogenesis in mice [28, 29]. In addition to screen drugs against the disease, it also provides the basic experimental tool to study the underlying cellular or molecular mechanisms of the disease. In the present study, the PTZ exerted its convulsant effects in both acute and chronic models that were profoundly reversed by tryptamine derivative HHL-6 (20 mg/kg dose). HHL-6 gave significant protection from mortality and forelimb and hind limb tonic extension in subcutaneous PTZ-induced seizure test. In addition, 20 mg/kg dose of HHL-6 was potent enough to retard the slow progression of seizure score when compared to PTZ-control group resulting in significant protective effect of HHL-6 on the development of kindling process. These observations were in accordance for the previous studies involving the compounds having tryptamine derivatives or indole rings in their structures [30–32]. Since PTZ most likely produces seizures by inhibiting GABA neurotransmission [33, 34] specifically acting as GABA_A_ receptor antagonist, the drugs preventing convulsions and seizures in PTZ-treated mice or increasing the latency to seizures may work either by enhancing GABA activity or antagonizing glutamate neurotransmission. Since HHL-6 effectively retards seizures in PTZ-induced seizure model and development of epileptogenesis in PTZ-kindled mice, therefore we suggest that it might be acting through enhancing GABA activity. However, further confirmation will be verified by performing studies on GABA receptors levels or by doing molecular studies involving gene expression for GABA.

In addition to these seizure related behavioral changes, there are various underlying cellular and molecular factors which are reported to be involved in the process of epileptogenesis such as c-Fos and BDNF [35–38]. The immunohistochemical analysis of the brain sections prepared from the kindled mice brain revealed that experimentally induced epilepsy elicited collateral expression of c-Fos protein in amygdala, cortex, hippocampus, and thalamus. The densitometry data of hippocampal CA1–CA3 regions were further analyzed using ImageJ analysis. Presently, we report that the tryptamine derivative HHL-6 and the reference drug diazepam greatly diminished the c-Fos protein expression showing no difference within these two groups. Our results further conform to the studies suggesting the proposed value of c-Fos in the modification in structure and function of neurons in mammalian nervous system to help define those brain structures that become activated by stimulation [39, 40]. Since, in earlier studies, it has also been reported that a single injection of PTZ (45 mg/kg) to mice induced c-Fos expression in several brain regions including the cerebral cortex and hippocampus [41]. Therefore, it has been suggested that induction of c-Fos expression might play an important role in the development of seizures as well as in excitatory amino-acid induced toxicity [42]. Hence, one of the purposes of this study was to determine whether the kindling induced overexpression of c-Fos protein was fundamentally modulated by our test compound HHL-6 or not. Our results show concordance approach to previous studies involving c-Fos overexpression in different models of neuronal stimulation and epilepsy [43, 44]. Interestingly, our test compound HHL-6 significantly reduces the expression of c-Fos in PTZ-treated mice as compared to normal control group providing potential antiepileptic effect.

Besides c-Fos, we also found a marked difference in the BDNF expression in the brain samples from normal control and PTZ-kindled control groups. Evidences demonstrate that epilepsy influences the expression of BDNF both at mRNA and BDNF protein levels [45, 46] probably due to modulation of excitatory and inhibitory neurotransmission [47]. Furthermore, studies have suggested that increased production of endogenous BDNF protein after a brain insult may be an important mediator of the cellular events that lead to the development of spontaneous seizures or epileptogenesis [48, 49]. Additional evidence puts forward the suggestion that limbic seizures can induce 4- to 10-fold increases in BDNF expression [50] and this increased expression of BDNF not only is associated with increased seizure severity but also induces various plastic changes in hippocampal neurons [51, 52] through activation of its receptor TrkB [53]. Moreover, it was reported that BDNF is a must for signaling through TrkB receptors for kindling [54] along with the fact that suppression of BDNF suppresses epileptogenesis [55]. Induction of c-Fos and BDNF is also preceded by activation of second messenger cascades like the MAP-kinase pathway [56] most likely resulting in the phosphorylation and activation of CREB protein [57]. All of these studies are in line with our results that further confirm this upregulation of BDNF protein in the kindled brain samples. Interestingly, our test compound was found to reduce the expression of BDNF which was comparable to the normal control group suggesting its probable antiepileptic action. The speculation that BDNF might regulate its own synthesis [58] leading to reduced BDNF signaling through downregulated TrkB receptors [59, 60] also supports our data. On the other hand, an additional piece of evidence establishes that TrkB is required for limbic epileptogenesis but not BDNF [61] suggesting the ligand independent activation of TrkB receptors [62, 63]. Therefore, next we will test the HHL-6 effects on the TrkB receptors to further identify the exact role of BDNF or its receptors (TrkA and TrkB) in the process of epileptogenesis.

## 5. Conclusion

Our study ultimately gives clear indication of significant retardation in the process of epileptogenesis by HHL-6 *in vivo* both in acute and chronic models. In addition, alteration in c-Fos and BDNF expressions in different brain regions provides a sensitive measure to elicit kindling behavior in mice and HHL-6 greatly modulates this overexpression suggesting its effective anticonvulsant and antiepileptogenic effect. This further emphasizes the need to confirm the activation of different neuronal pathways with different types of seizures by the help of additional molecular studies on receptor level.

## Figures and Tables

**Table 1:** Treatment regime followed for subcutaneous-PTZ-induced seizure test.

Group	Treatment	Dose
GI	Normal control	0.2 mL of 0.9% saline
GII	PTZ-control	90 mg/kg
GIII	Diazepam + PTZ	7.5 mg/kg + 90 mg/kg
GIV	HHL-6 + PTZ	20 mg/kg + 90 mg/kg

**Table 2:** Treatment regime followed for PTZ-induced kindling test.

Group	Treatment (administered on alternate days)	Dose
GI	Normal control	0.2 mL of 0.9% saline
GII	PTZ-control	50 mg/kg
GIII	Diazepam + PTZ	7.5 mg/kg + 50 mg/kg
GIV	HHL-6 + PTZ	20 mg/kg + 50 mg/kg

**Table 3:** Effect of HHL-6 on the duration and inhibition of seizure in scPTZ-induced seizure in mice.

Group	Dose	Onset of jerks (sec)	Rearing and falling (sec)
GI (Normal control)	0.2 mL/kg	0	0
GII (PTZ-control)	90 mg/kg	190	640
GIII (Diazepam + PTZ)	7.5 mg/kg	0	0
GIV (HHL-6 + PTZ)	20 mg/kg	460	0

The HHL-6 and diazepam were injected intraperitoneally 30 min before the administration of subcutaneous pentylenetetrazole (90 mg/kg) in their respective groups. Values are presented as mean for the duration of tonic seizure for 6 mice.

**Table 4:** Fluorescence intensity of c-Fos (arbitrary unit) in various regions of brain from normal and kindled mice.

Brain regions	Experimental group			
	Normal control	PTZ-control	DZP + PTZ	HHL-6 + PTZ
Amygdala	12.23 ± 2.9	38.17 ± 5.08	14.94 ± 1.99	15.21 ± 2.3
Cortex	16.31 ± 1.24	40.69 ± 8.2	17.28 ± 3.6	18.24 ± 3.2
Hippocampus	15.29 ± 2.3	44.27 ± 7.43	20.09 ± 3.77	16.92 ± 2.75
Thalamus	12.58 ± 1.79	45.36 ± 3.70	18.33 ± 2.19	15.58 ± 3.74

Significant increase in the expression of c-Fos protein in the brain sections of control PTZ-kindled mice (*P* < 0.001) was centered especially in the hippocampal region followed by the thalamus. The HHL-6 treatment however reduces the expression of c-Fos in all four selected regions, that is, amygdala, cortex, hippocampus, and thalamus.

## References

[1] Fisher RS, Van Emde Boas W, Blume W (2005). Epileptic seizures and epilepsy: definitions proposed by the International League Against Epilepsy (ILAE) and the International Bureau for Epilepsy (IBE). *Epilepsia*.

[2] World Health Organization (2001). *Epilepsy: Epidemiology, Aetiology and Prognosis*.

[3] Sato T, Yamada N, Morimoto K, Uemura S, Kuroda S (1998). A behavioral and immunohistochemical study on the development of perirhinal cortical kindling: a comparison with other types of limbic kindling. *Brain Research*.

[4] Li J, Shen H, Naus CCG, Zhang L, Carlen PL (2001). Upregulation of gap junction connexin 32 with epileptiform activity in the isolated mouse hippocampus. *Neuroscience*.

[5] Gorji A, Madeja M, Straub H, Köhling R, Speckmann E-J (2001). Lowering of the potassium concentration induces epileptiform activity in guinea-pig hippocampal slices. *Brain Research*.

[6] Pitkänen A, Lukasiuk K (2009). Molecular and cellular basis of epileptogenesis in symptomatic epilepsy. *Epilepsy and Behavior*.

[7] Löscher W (2002). Current status and future directions in the pharmacotherapy of epilepsy. *Trends in Pharmacological Sciences*.

[8] Pitkänen A, Kharatishvili I, Karhunen H (2007). Epileptogenesis in experimental models. *Epilepsia*.

[9] Akula KK, Dhir A, Bishnoi M, Kulkarni SK (2007). Effect of systemic administration of adenosine on brain adenosine levels in pentylenetetrazol-induced seizure threshold in mice. *Neuroscience Letters*.

[10] Rattka M, Brandt C, Bankstahl M, Bröer S, Löscher W (2011). Enhanced susceptibility to the GABA antagonist pentylenetetrazole during the latent period following a pilocarpine-induced status epilepticus in rats. *Neuropharmacology*.

[11] Sato M (1982). Kindling: an experimental model of epilepsy. *Psychiatry and Clinical Neurosciences*.

[12] Sato M, Racine RJ, McIntyre DC (1990). Kindling: basic mechanisms and clinical validity. *Electroencephalography and Clinical Neurophysiology*.

[13] Yoshii A, Constantine-Paton M (2010). Postsynaptic BDNF-TrkB signaling in synapse maturation, plasticity, and disease. *Developmental Neurobiology*.

[14] Ballarin M, Ernfors P, Lindefors N, Persson H (1991). Hippocampal damage and kainic acid injection induce a rapid increase in mRNA for BDNF and NGF in the rat brain. *Experimental Neurology*.

[15] Lähteinen S, Pitkänen A, Knuuttila J, Törönen P, Castrén E (2004). Brain-derived neurotrophic factor signaling modifies hippocampal gene expression during epileptogenesis in transgenic mice. *European Journal of Neuroscience*.

[16] Larmet Y, Reibel S, Carnahan J, Nawa H, Marescaux C, Depaulis A (1995). Protective effects of brain-derived neurotrophic factor on the development of hippocampal kindling in the rat. *NeuroReport*.

[17] Simonato M, Molteni R, Bregola G (1998). Different patterns of induction of FGF-2, FGF-1 and BDNF mRNAs during kindling epileptogenesis in the rat. *European Journal of Neuroscience*.

[18] Herrera DG, Robertson HA (1996). Activation of c-fos in the brain. *Progress in Neurobiology*.

[19] Curran T, Morgan JI (1995). Fos: an immediate-early transcription factor in neurons. *Journal of Neurobiology*.

[20] Kovács KJ (1998). c-Fos as a transcription factor: a stressful (re)view from a functional map. *Neurochemistry International*.

[21] Sagar SM, Sharp FR, Curran T (1988). Expression of c-fos protein in brain: metabolic mapping at the cellular level. *Science*.

[22] Dragunow M, Robertson HA (1988). Localization and induction of c-fos protein-like immunoreactive material in the nuclei of adult mammalian neurons. *Brain Research*.

[23] Kiessling M, Gass P (1993). Immediate early gene expression in experimental epilepsy. *Brain Pathology*.

[24] Iryo Y, Matsuoka M, Igisu H (2000). Suppression of pentylenetetrazol-induced seizures and c-fos expression in mouse brain by L-carnitine. *Journal of Occupational Health*.

[25] Humpel C, Wetmore C, Olson L (1993). Regulation of brain-derived neurotrophic factor messenger RNA and protein at the cellular level in pentylenetetrazol-induced epileptic seizures. *Neuroscience*.

[26] Racine RJ (1972). Modification of seizure activity by electrical stimulation: II. Motor seizure. *Electroencephalography and Clinical Neurophysiology*.

[27] Racine R, Tuff L, Zaide J (1975). Kindling, unit discharge patterns and neural plasticity. *Canadian Journal of Neurological Sciences*.

[28] Faingold CL, Jobes PC, Laird HE (1987). Seizures induced by convulsant drugs. *Neurotransmitters and Epilepsy*.

[29] Mandhane SN, Aavula K, Rajamannar T (2007). Timed pentylenetetrazol infusion test: a comparative analysis with s.c.PTZ and MES models of anticonvulsant screening in mice. *Seizure*.

[30] Oliveira FA, De Almeida RN, Sousa MDFV, Barbosa-Filho JM, Diniz SA, De Medeiros IA (2001). Anticonvulsant properties of *N*-salicyloyltryptamine in mice. *Pharmacology Biochemistry and Behavior*.

[31] Oliveira FDA, E Silva DA, Quintans LJ (2006). Synthesis and structural characterization of *N*-benzoyltryptamine and its new analogue *N*-salicyloyltryptamine, a potential anticonvulsant agent. *Journal of the Chilean Chemical Society*.

[32] Altintaş H, Ateş Ö, Uydeş-Doğan BS (2006). Synthesis and evaluation of antimicrobial and anticonvulsant activities of some new 3-[2-(5-aryl-1,3,4-oxadiazol-2-yl/4-carbethoxymethylthiazol-2-yl)imino-4-thiazolidinon-5-ylidene]-5-substituted/nonsubstituted 1H-indole-2-ones and investigation of their structure-activity relationships. *Arzneimittel-Forschung*.

[33] Gale K (1992). Role of GABA in the genesis of chemoconvulsant seizures. *Toxicology Letters*.

[34] De Sarro G, Palma E, Costa N (2000). Effects of compounds acting on GABA(B) receptors in the pentylenetetrazole kindling model of epilepsy in mice. *Neuropharmacology*.

[35] Morgan JI, Cohen DR, Hempstead JL, Curran T (1987). Mapping patterns of c-fos expression in the central nervous system after seizure. *Science*.

[36] Burazin TCD, Gundlach AL (1996). Rapid and transient increases in cellular immediate early gene and neuropeptide mRNAs in cortical and limbic areas after amygdaloid kindling seizures in the rat. *Epilepsy Research*.

[37] Mhyre TR, Applegate CD (2003). Persistent regional increases in brain-derived neurotrophic factor in the flurothyl model of epileptogenesis are dependent upon the kindling status of the animal. *Neuroscience*.

[38] Clark M, Post RM, Weiss SRB, Cain CJ, Nakajima T (1991). Regional expression of c-fos mRNA in rat brain during the evolution of amygdala kindled seizures. *Molecular Brain Research*.

[39] Labiner DM, Butler LS, Cao Z, Hosford DA, Shin C, McNamara JO (1993). Induction of c-fos mRNA by kindled seizures: complex relationship with neuronal burst firing. *Journal of Neuroscience*.

[40] Erdtmann-Vourliotis M, Riechert U, Mayer P, Grecksch G, Höllt V (1998). Pentylenetetrazole (PTZ)-induced c-fos expression in the hippocampus of kindled rats is suppressed by concomitant treatment with naloxone. *Brain Research*.

[41] Giulia D, Francesca V, Sandra S (2006). A molecular study of hippocampus in dogs with convulsion during canine distemper virus encephalitis. *Brain Research*.

[42] Szyndler J, Maciejak P, Turzyńska D (2009). Mapping of c-Fos expression in the rat brain during the evolution of pentylenetetrazol-kindled seizures. *Epilepsy and Behavior*.

[43] Kasof GM, Mandelzys A, Maika SD, Hammer RE, Curran T, Morgan JI (1995). Kainic acid-induced neuronal death is associated with DNA damage and a unique immediate-early gene response in c-fos-lacZ transgenic rats. *Journal of Neuroscience*.

[44] Chiasson BJ, Hong MGL, Robertson HA (1997). Putative roles for the inducible transcription factor c-fos in the central nervous system: studies with antisense oligonucleotides. *Neurochemistry International*.

[45] Nawa H, Carnahan J, Call C (1995). BDNF protein measured by a novel enzyme immunoassay in normal brain and after seizure: partial disagreement with mRNA levels. *European Journal of Neuroscience*.

[46] Numakawa T, Suzuki S, Kumamaru E, Adachi N, Richards M, Kunugi H (2010). BDNF function and intracellular signaling in neurons. *Histology and Histopathology*.

[47] Binder DK, Croll SD, Gall CM, Scharfman HE (2001). BDNF and epilepsy: too much of a good thing?. *Trends in Neurosciences*.

[48] Wang Y, Qi J-S, Kong S (2009). BDNF-TrkB signaling pathway mediates the induction of epileptiform activity induced by a convulsant drug cyclothiazide. *Neuropharmacology*.

[49] Lindholm D, Castren E, Berzaghi M, Blochl A, Thoenen H (1994). Activity-dependent and hormonal regulation of neurotrophin mRNA levels in the brain—implications for neuronal plasticity. *Journal of Neurobiology*.

[50] Elmer E, Kokaia M, Kokaia Z, Ferencz I, Lindvall O (1996). Delayed kindling development after rapidly recurring seizures: relation to messy fiber sprouting and neurotrophin, GAP-43 and dynorphin gene expression. *Brain Research*.

[51] Isackson PJ, Huntsman MM, Murray KD, Gall CM (1991). BDNF mRNA expression is increased in adult rat forebrain after limbic seizures: temporal patterns of induction distinct from NGF. *Neuron*.

[52] Croll SD, Suri C, Compton DL (1999). Brain-derived neurotrophic factor transgenic mice exhibit passive avoidance deficits, increased seizure severity and in vitro hyperexcitability in the hippocampus and entorhinal cortex. *Neuroscience*.

[53] Wetmore C, Olson L, Bean AJ (1994). Regulation of brain-derived neurotrophic factor (BDNF) expression and release from hippocampal neurons is mediated by non-NMDA type glutamate receptors. *Journal of Neuroscience*.

[54] Gottschalk W, Pozzo-Miller LD, Figurov A, Lu B (1998). Presynaptic modulation of synaptic transmission and plasticity by brain- derived neurotrophic factor in the developing hippocampus. *Journal of Neuroscience*.

[55] He X-P, Minichiello L, Klein R, McNamara JO (2002). Immunohistochemical evidence of seizure-induced activation of trkB receptors in the mossy fiber pathway of adult mouse hippocampus. *Journal of Neuroscience*.

[56] Gass P, Kiessling M, Bading H (1993). Regionally selective stimulation of mitogen activated protein (MAP) kinase tyrosine phosphorylation after generalized seizures in the rat brain. *Neuroscience Letters*.

[57] Herdegen T, Blume A, Buschmann T (1997). Expression of activating transcription factor-2, serum response factor and camp/ca response element binding protein in the adult rat brain following generalized seizures, nerve fibre lesion and ultraviolet irradiation. *Neuroscience*.

[58] Heinrich C, Lähteinen S, Suzuki F (2011). Increase in BDNF-mediated TrkB signaling promotes epileptogenesis in a mouse model of mesial temporal lobe epilepsy. *Neurobiology of Disease*.

[59] Kokaia M, Ernfors P, Kokaia Z, Elmer E, Jaenisch R, Lindvall O (1995). Suppressed epileptogenesis in BDNF mutant mice. *Experimental Neurology*.

[60] Lähteinen S, Pitkänen A, Saarelainen T, Nissinen J, Koponen E, Castrén E (2002). Decreased BDNF signalling in transgenic mice reduces epileptogenesis. *European Journal of Neuroscience*.

[61] Frank L, Ventimiglia R, Anderson K, Lindsay RM, Rudge JS (1996). BDNF down-regulates neurotrophin responsiveness, TrkB protein and TrkB mRNA levels in cultured rat hippocampal neurons. *European Journal of Neuroscience*.

[62] Sommerfeld MT, Schweigreiter R, Barde Y-A, Hoppe E (2000). Down-regulation of the neurotrophin receptor TrkB following ligand binding: evidence for an involvement of the proteasome and differential regulation of TrkA and TrkB. *Journal of Biological Chemistry*.

[63] He X-P, Kotloski R, Nef S, Luikart BW, Parada LF, McNamara JO (2004). Conditional deletion of TrkB but not BDNF prevents epileptogenesis in the kindling model. *Neuron*.

